# A new approach to skin extravasation injury management during the neonatal period

**DOI:** 10.1186/s12887-022-03511-y

**Published:** 2022-07-26

**Authors:** Setareh Sagheb, Sayyed Ourmazd Mohseni, Ameneh Lamsehchi

**Affiliations:** 1 Department of Neonatology, Shariati Hospital, Tehran University of Medical Sciences, Tehran, Iran; 2 Maternal, Fetal & Neonatal Research Center, Tehran University of Medical Sciences, Tehran, Iran; 3 Herman Ostrow School of Dentistry, University of Southern California, Los Angeles, CA USA

**Keywords:** Extravasation, Neonate, Hyaluronidase, Fibrinolysin, Phenytoin ointment

## Abstract

**Background:**

To identify a standard protocol for managing extravasation injuries in neonates.

**Methods:**

We recruited all the neonates with extravasation wounds from the neonatal intensive care unit of Shariati hospital, Tehran, Iran, between October 2018 and October 2020. Sixteen patients with grade 3–4 extravasation were evaluated in this retrospective study. All grade 3 and 4 extravasation wounds were injected with hyaluronidase at 5 points of the wound circle; the procedure was repeated every 5 min at different points in a smaller circle to the core. The wound was then covered with a warm compress for 24 h. Twenty-four hours after injection, the cover was changed twice a day with normal saline irrigation. Fibrinolysin ointment was applied on top of the wound. The ulcer was then dressed with phenytoin ointment until healing.

**Results:**

Out of 16 neonates who were followed up, 10 of them were male, with the average birth weight being 1.37 (range 1.05–3.75) kg. The mean (± SD) wound healing duration was 13.12 (± 6) (range: 7–29) days. Factors including the cannulation duration before the appearance of the lesion (R:0.2, *P* = 0.2), birth weight (*R* = -.37, *P* = 015), and extravasated substances (*p* = 0.2) were not associated with the duration of hospital stay. The only exception to this trend is the wound size factor of 7.31(± 7.45) (*R* = .83, *P* < 0.001). Continuous and categorical variables were summarized as mean (SD) and proportions, respectively, and the Kruskal–Wallis test and Spearman correlation coefficients were used.

**Conclusions:**

Limited evidence exists on the effects of different protocols on extravasation management in neonates in the NICU. We recommend our method as a standard protocol in NICU for high-stage extravasated lesions because of the shorter duration of healing, non-invasive nature of this procedure, and lack of side effects or surgical involvement.

## Background

Extravasation is described as unintentionally administering drugs to the perivascular or subcutaneous tissues from the intravenous line. This leakage of agents can injure surrounding tissue, tendon, nerve and may cause inflammation, infection, and ulceration. Ultimately, tissue necrosis, muscle contracture, and functional sequela may result [1]. The degree of tissue injury depends on factors such as drug characteristics, level of toxicity, the insertion site of the intravascular cannula, and the amount of the drug infiltrated. The mechanism of necrosis by drug infiltration to the tissue is unknown, but it seems to be associated with PH, osmolality, and separation of ions in extravasated substances [2]. The incidence rate of extravasation ranges from 11 to 70% in children with intravenous catheters. However, other papers report a lower prevalence of extravasation in children [3–7].

Moreover, the rate of 70% refers to neonates with intravenous therapy [4, 8]. Extravasation injury has different underlying factors, including deracination of intravenous line access, vascular fragility, or reverse run of drugs to the interstitial space due to vein blockage [2, 4, 9, 10]. Extravasation is categorized into four stages:

- Stage 1: area of infiltration has pain but is not bulged or erythematous.
- Stage 2: area of infiltration has pain, has a little bulging and erythema, and capillary refilling is quick and normal.
- Stage 3: area of infiltration has pain and noticeable bulging; it has a good pulse and regular capillary refilling, but it is cold in taping skin.
- Stage 4: infiltration area has pain, very noticeable bulging, a weak or absent pulse, a prolonged capillary refill time (more than 4 s), and cutaneous necrosis [11, 12]. Usually, patients with stages 1 and 2 are managed by conservative dressings. More severe injuries (stages 3 and 4) typically require interventions like hyaluronidase injections, aspirations or drainage of abscesses, and debridement necrosis [13]. The common consensus is to start the treatment as soon as possible, and the first step is to cease infusion immediately. More actions in extravasation management rely on the severity and the type of fluid, which is extravasated and based on experts’ advice and hospital protocols. The major objective is to relieve pain, improve tissue perfusion, heal the wound and prevent complications. [14]. The best strategy for managing extravasation is prevention as well as checking intravenous line access repeatedly [9]. The current approach to neonatal peripheral extravasation appears to be conservative management, which includes removing an intravenous catheter, limb elevation, applying warmth on an opposite limb( reflex vasodilation), elastic bandaging, manual lymphatic drainage immobilization, and local dressing [14, 15]. Many treatments and methods are discussed, such as antidote injection for neutralizing vesicant solution of chemotherapy extravasation [16]. Furthermore, applying topical nitroglycerin (TNG) ointment on the tissue ischemic injuries of neonates with 76.0% complete recovery may be effective [15]. TNG is useful in the extravasation of vassopressors due to its local vasodilatory effect [17]. In the case of irritating or vesicant fluid extravasations, conservative therapy may be ineffective, and therefore antidote administration and surgical management may be required [14]. Hyaluronidase is one of the vital therapeutic factors for treatment. Hyaluronidase breaks down glycosaminoglycan and hyaluronic acid and raises the clearance of the drug. However, there is still controversy over the standard dosage and appropriate injection [18]. It seems that the injection of 15 units of this agent is suitable for extravasation in children; however, the dosage has been reported to have a broad range (15–1500 units with Saline flush) [5, 18]. Although using a cold or warm compress for extravasation management is controversial, applying a warm compress with hyaluronidase can cause a rise in blood circulation and assist dispersal of the vesicant away from the extravasation site to reduce tissue damage [17]. There is no global consensus for extravasation management, despite the potential risk of severe complications during hospitalization. Additionally, every hospital approaches extravasation with their local guidelines [1, 2, 19]. To date, appropriate protocols for managing extravasation injuries are the main topic of debate.

## Methods

We gathered all the neonates with extravasation wounds from the neonatal intensive care unit of Shariati hospital, Tehran, Iran, between October 2018 and October 2020. Sixteen patients with grade 3–4 extravasation were evaluated in this retrospective study. The Medical Ethics Committee of Tehran University of Medical Sciences approved this study design (IR.TUMS.MEDICINE.REC.1399.091), and informed consent forms were filled out and collected.

Inclusion criteria were the clinical appearance of stage 3 and 4 extravasation lesions (notable bulging, discoloration of the skin, delayed capillary filling, ischemia, necrosis, and low pulse). Exclusion criteria are stage 1 and 2 extravasations, infection, acutely inflamed area, dopamine, or alpha agonist agents.

Twenty neonates with these criteria were included, and four were excluded due to inflamed and infected lesions and dopamine-induced lesions.

Sex, age, size, site of intravenous extravasation, fluid type, and wound healing period were analyzed. There was a definite protocol for extravasation in the neonatal intensive care unit ( NICU). All grade 3 and 4 extravasation wounds were injected with hyaluronidase (Hyalase 1500 IU powder diluted with 10 ml distilled water). An insulin syringe needle with a gauge range of 28–31 was utilized for the injections. 0.2 ml of the solution mentioned above was injected into 5 points of the wound circle; the procedure was repeated every 5 min at different points in a smaller circle to the core. We used a warmer with 37.5 °C to warm the hot water bag to this degree, warming a sterile gauze to dress the wound during a 5-min interval between every five injections. Twenty-four hours after injection, the cover was changed twice a day with normal saline irrigation. Fibrinolysin ointment was applied on top of the wound. The ulcer was then dressed with phenytoin ointment until healing (phenytoin ointment (MEDIFARM) 1%—each 100 gr contain 1 g sodium phenytoin- and Fibrinolysin/Desoxyribonuclease ointment (WESTAGEN PARS). Moreover, during the injection procedure, we used non-pharmacological methods such as a pacifier covered in breast milk or sucrose as a nonnutritive sucking and postured neonates to flexion positions to manage pain.

## Results

Out of 20 Infants who were screened for eligibility, four individuals were excluded, and eventually, 16 cases were successfully followed up. The mean (SD) gestational age and cannulation duration when the lesion appeared were 32 (2.62) weeks and 7.87(2.41) days, respectively. The average (median) birth weight was 1.37 (range 1.05–3.75) kg, and 10(62.5) of the infants were male. The mean (SD) wound healing duration was 13.12 (± 6) (range: 7–29) days (Table 1).

**Table 1:** Patients’ demographics

Characteristic	Value: Mean(± SD)
Male:female(%)	10(62.5%):6(37.5%)
Birth weight (kg)	1.70(± 0.83)
Gestational age(week)	32.1(± 2.62)
Cannulation time before lesion appeared (day)	7.87(± 2.41)
Wound healing duration (day)	13.12(± 6)
Lesion size (cm^2^)	7.31(± 7.45)

The site of intravenous cannulation and the substances involved in extravasation events are shown in Table 2.

**Table 2:** Characteristics of extravasation

Location	**No. of patients (%)**
Dorsum of hand	9(56.25)
Distal lower limb	5(31.25)
Wrist	2(12.5)
Extravasated substance	
Total parenteral nutrition (Intralipid)	2(12.5)
Calcium chloride	9(56.3)
12.5% Dextrose water	5 (31.3)

Factors including the cannulation duration before the appearance of the lesion (R:0.2, *P* = 0.2), birth weight (*R* = -0.37, *P* = 015), and extravasated substances (*p* = 0.2) were not associated with the duration of hospital stay. The only exception to this trend is the wound size factor (*R* = 0.83, *P* < 0.001).

Data analysis was undertaken using SPSS version 21 (SPSS, Inc., Chicago, IL). Continuous and categorical variables were summarized as mean (SD) and proportions, respectively, and the Kruskal–Wallis test and Spearman correlation coefficients were used.

**Fig. 1:**
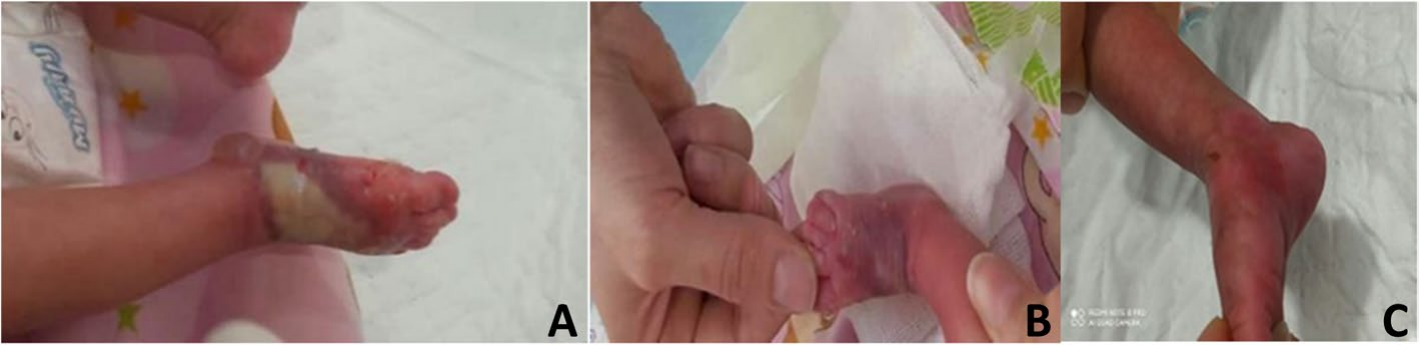
**A** Stage four extravasation lesion in the dorsum of the right foot. **B** The result after 24 h. **C** Clinical characteristics had disappeared during four days follow up

**Fig. 2:**
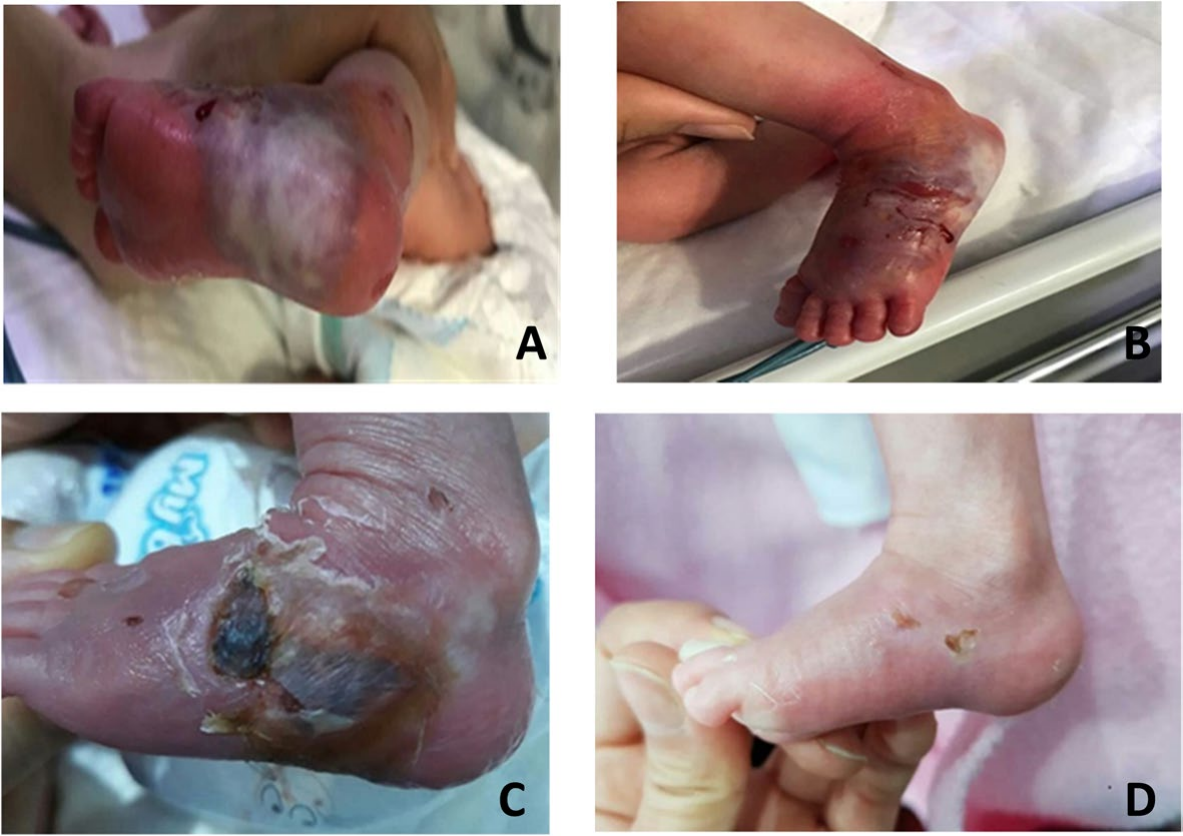
**A**, **B** Stage four extravasation lesion in the left foot’s dorsal and plantar surface. **C** The result after 24 h. **D** clinical characteristic had disappeared 30 days follow up during admission

None of the wounds developed any complications, and surgical management was not required. During the time of admission, all of the lesions completed the healing process, and the patients were discharged without sequel (Figs. 1– 2).

## Discussion

Extravasation lesions are more common in neonates and infants than in adults. The leading underlying causes are the fragility and small diameter of the vessels and the lack of subcutaneous tissue [20]. There are no standard strategies for approaching extravasations in neonates and children worldwide to the best of our knowledge. Extravasation management diversifies into conservative to invasive methods. First, prevention and education programs for nurses play a crucial role in reducing the peripheral extravasation rate. This is highly effective if implemented periodically [21]. This article describes our hospital protocol in extravasation management.

Regarding hyaluronidase, several papers reported the successful intradermal use of the solution as described in our study. However, the Food and Drug Administration (FDA) has not yet approved this indication. Furthermore, randomized clinical trials for the efficacy of this method have not been performed [18]. Rowllet et al. reported human hyaluronidase injection for extravasation of contrast media in a circle around the wound, which led to improvements during 4 h [22]. Fox et al. illustrated wound enhancement after injection of hyaluronidase following amiodarone extravasation without any complications [23].

Moreover, Kostogloudis et al. claimed that gestational age is associated with skin necrosis occurrence; most of these lesions appeared in neonates of 26 weeks or less [20]. Our data, however, showed that the mean (SD) gestational age is 32.1 weeks. The severity of the wound depends on many factors. As explained above, one of the key factors is the type of extravasant. Despite previous studies that illustrated total parenteral nutrition (TPN) as the cause of most lesions after extravasation [13, 20], our study found that calcium chloride was responsible for most high-stage wounds ( 56.3%). Less common damages were related to TPN.

Similar to previous surveys, our study demonstrates that up to 70% of all extravasation injuries occur in the upper limbs ( 68.75% to 70%). This may be due to easier access in the upper limb than lower limbs [3, 4, 13]. In the present research, wound healing duration was 13.12 ± 6 days, which was associated with the wound size factor (*R* = 0.83, *P* < 0.001). We believe that our healing duration in stage 3,4 extravasation lesions was shorter than the one in Cho et al.’s previous study; (13.12 days to an average of 18.7 days) despite having more extensive wound sizes ( 7.31cm2 to 2.24 cm2). Based on our data, the cannulation duration at the extravasation injury was longer than the one in the survey conducted by Cho et al. (7.87 days to 3.2) [13]. This proposes a precise observation of the intravenous line site to prevent injury, at least during this time.

In the present study, we strongly recommend a repetitive modified hyaluronidase injection method soon after extravasation and warm compression for 24 h, as recommended in a previous study carried out by Firat et al. [24]. Furthermore, changing the wound cover twice a day with normal saline irrigation along with a fibrinolysin covering and phenytoin ointment until healing is highly recommended. The advantages of this method include not requiring surgical methods and anesthesia as well as lack of complications. Notably, this is a simple procedure, and cosmetic and functional outcome is guaranteed.

## Conclusion

Prevention and management guidelines of extravasation injuries should be taught to the healthcare team to manage this complication as soon as it occurs. The intravenous line site should be monitored at least seven days after cannulation in the neonatal unit. We highly recommend our method as a standard protocol in NICU for high-stage extravasated lesions because of the shorter duration of healing, non-invasive nature of this procedure, and lack of side effects or surgical involvement.

## Data Availability

All data, including patients’ medical records, images, and laboratory data, are kept in our hospital for a minimum of 5 years based on the local regulations.

## Abbreviations

NICU: Neonatal intensive care unit
FDA: Food and Drug Administration
TPN: Total parenteral nutrition
TNG: Topical nitroglycerin
